# Association of bone mineral density with prediabetes risk among African-American and European-American adult offspring of parents with type 2 diabetes

**DOI:** 10.3389/fendo.2022.1065527

**Published:** 2023-01-05

**Authors:** Zhao Liu, Peace Asuzu, Avnisha Patel, Jim Wan, Sam Dagogo-Jack

**Affiliations:** ^1^ Department of Medicine, Division of Endocrinology, Diabetes and Metabolism, University of Tennessee Health Science Center, Memphis, TN, United States; ^2^ Department of Preventive Medicine, University of Tennessee Health Science Center, Memphis, TN, United States

**Keywords:** bone mineral density, impaired fasting glucose, impaired glucose tolerance, prospective study, race/ethnicity

## Abstract

**Introduction:**

Type 2 diabetes mellitus (T2DM) is associated with alterations in bone mineral density (BMD), but association between prediabetes and BMD is unclear.

**Methods:**

We analyzed BMD among the initially normoglycemic participants in the Pathobiology of Prediabetes in a Biracial Cohort (POP-ABC) study in relation to incident prediabetes during 5 years of follow-up.

**Results and Discussion:**

A total of 343 participants (193 Black, 150 White) underwent DEXA during Year 1 of POP-ABC and were followed quarterly for 5 years. The mean age was 44.2 ± 10.6 years; BMI was 30.2 ± 7.23 kg/m^2^. At baseline, the mean BMD was 1.176 ± 0.135 g/cm^2^ (1.230 ± 0.124 g/cm^2^ in men vs. 1.154 ± 0.134 g/cm^2^ in women, P<0.0001; 1.203 ± 0.114 g/cm^2^ in Black vs. 1.146 ± 0.150 g/cm^2^ in White participants, P=0.0003). During 5 years of follow-up, 101 participants developed prediabetes and 10 subjects developed T2DM (progressors); 232 were nonprogressors. Progressors to prediabetes had numerically higher baseline BMD and experienced lower 1-year decline in BMD (P<0.0001) compared with nonprogressors. From Kaplan-Meier analysis, the time to 50% prediabetes survival was 2.15 y among participants in the lowest quartile of baseline BMD, longer than those in higher quartiles (1.31 – 1.41 y). Values for BMD correlated inversely with age and adiponectin levels, and positively with BMI. In logistic regression analysis, BMD z score significantly predicted incident prediabetes: more negative BMD z scores were associated with decreased incident prediabetes (odds ratio 0.598 [95% confidence interval 0.407 - 0.877], P=0.0085), after controlling for age, BMI, change in BMI, ethnicity, blood glucose and adiponectin.

**Conclusions:**

Among initially normoglycemic individuals, higher baseline BMD was associated with higher risk of incident prediabetes during 5 years of follow-up.

## 1 Introduction

Diabetes mellitus appears to exhibit a complex relationship with bone health. Cross-sectional studies have reported lower bone mineral density (BMD) in people with type 1 diabetes mellitus (T1DM) (1–3) but similar or higher BMD in those with type 2 diabetes (T2DM) (4–6) compared with healthy control subjects. In one study, the mean BMD in patients with T2DM was ~10% higher than that of age-matched individuals without diabetes (4). In a meta-analysis of 15 observational studies with a pooled population of 3,437 T2DM patients and 19,139 controls, BMD was significantly higher by 0.04 g/cm^2^ at the femoral neck, 0.06 g/cm^2^ at the hip and 0.06 g/cm^2^ at the spine in T2DM patients versus controls (5). The mechanisms for the higher BMD in people with T2DM are not known precisely but may be related to adiposity, hyperglycemia, or hyperinsulinemia (6, 7). The Rotterdam study found that patients with inadequately controlled T2DM had higher BMD compared with healthy subjects or patients with adequately controlled T2DM (6).

Paradoxically, the normal or higher BMD observed in people with T2DM is not associated with the expected decrease in the risk of fracture. In fact, increased fracture risk may be higher in people with diabetes versus healthy control (4, 6–8). In the prospective Japanese Nurses’ Health Study, among women 34-59 years old the incidence of hip fractures was six-fold higher in patients with T1DM and two-fold higher in those with T2DM compared with healthy subjects, after adjustments for body mass index (BMI), smoking, physical activity, menopausal status, postmenopausal hormone use, and daily intake of calcium, vitamin D and protein (8). Multiple factors than can contribute to increased fracture risk in people with diabetes include alterations in bone microstructure, increased cortical porosity, and reduced cortical density (8–13). Furthermore, insulin deficiency and low levels of IGF-1 in patients with type 1 diabetes impair osteoblast function, leading to low peak bone mass at a young age (10). Additional diabetes-related deleterious factors include formation of advanced glycation end products, inflammatory cytokines, osteocyte production of sclerostin, and bone microvascular disease (8–13). Finally, certain medications used for treating diabetes have been associated with alterations in bone metabolism and fracture risk (14).

We explored the ontogeny of the association between diabetes and increased BMD by studying normoglycemic individuals who developed prediabetes during prospective follow-up. We reasoned that a true biological association between increased bone mass and T2DM might be discernible at the more proximal stage of prediabetes. Among persons at genetic risk for T2DM, the transition from normoglycemia to diabetes often follows a predictable course through an intermediate stage of prediabetes, defined as impaired fasting glucose (IFG) and/or impaired glucose tolerance (IGT) (15–17). The Centers for Disease Control and Prevention estimates that approximately 96 million Americans aged 18 years and older have prediabetes (18). Unlike in patients with established diabetes, the relation between BMD and prediabetes has not been well studied. In one report, based on data from the U.S. National Health and Nutrition Examination Surveys (NHANES) from 2005 to 2014, adults 40 years of age or older with prediabetes had higher BMD but greater hip fracture risk compared with adults with normal glucose tolerance (19). In another cross-sectional study (based on NHANES 2005-2018 data), there was an increasing trend of BMD at the hip, femoral neck, and lumbar spine across the glycemic spectrum from normoglycemia, prediabetes, to diabetes in adults aged 40 years or older (20). However, these cross-sectional observations do not reveal the direction of the association between BMD and diabetes or prediabetes, nor do they permit causal inferences. Prospective studies are needed to demonstrate directionality and enable the identification of possible causal mechanisms of the association between bone mass and disorders of glucose metabolism.

The Pathobiology of Prediabetes in a Biracial Cohort (POP-ABC) study enrolled self-reported African American and European American adults with parental T2DM and assessed progression from normoglycemia to T2DM during for 5 years of follow-up (21–26). The primary results of the POP-ABC study, which showed no ethnic disparity in the incidence of prediabetes among people with similar parental history of T2DM, identified baseline weight, insulin sensitivity, insulin secretion and inflammatory markers as significant associations of prediabetes risk (26). In the present *post-hoc* analysis, we examined the association between BMD at enrollment and incident prediabetes risk in the POP-ABC study. We further assessed the relationship between BMD and several demographic, biochemical, and glucoregulatory variables, to explore potential mechanisms for any association between BMD and prediabetes. The prospective design of the POP-ABC study enabled us to track initially normoglycemic individuals until the occurrence of IFG or IGT and determine whether baseline BMD is associated with such an outcome. Furthermore, by studying a normoglycemic population, we avoided the confounding effects of anti-diabetes medications on bone metabolism that plagued cross-sectional studies of people with established diabetes.

## 2 Materials and methods

### 2.1 Study subjects

The study subjects were participants in the POP-ABC study (21–23). Eligible for enrolment in the POP-ABC study were healthy, normoglycemic adults aged 18–65 years who self-reported as being of non-Hispanic white (European American) or non-Hispanic black (African American) ancestry and had one or both biological parents with T2DM. The standard 75-gram oral glucose tolerance test (OGTT) was used to screen prospective participants and those with normal fasting plasma glucose (FPG, <100 mg/dL [5.6 mmol/L]) and normal glucose tolerance (NGT, 2-hour plasma glucose [2hPG] <140 mg/dL [7.8 mmol/L]), based on American Diabetes Association criteria, were enrolled (15, 24). Excluded from participation were individuals with a history of diabetes, those taking glucocorticoids or medications known to alter body weight, blood glucose or bone metabolism, or persons enrolled in behavioral weight loss programs or having a history of bariatric surgery. Individuals self-reported their race/ethnicity, based on the 1990 US Census questionnaire (25). The University of Tennessee Institutional Review Board approved the study protocol. All participants gave written informed consent before initiation of the study, which was conducted at the University of Tennessee General Clinical Research Center (GCRC).

### 2.2 Assessments

Participants made outpatient visits to the GCRC after an overnight fasting at baseline and every 3 months during 5 years of follow-up. Assessments at baseline included anthropometric measurements (weight, height, BMI, waist circumference), body composition (total fat mass, trunk fat mass) and bone densitometry by dual-energy x-ray absorptiometry (DEXA) (Hologic Discovery A80044A, Hologic Inc., Bedford, MA), OGTT, and biochemistries (21–23). Assessments during year one included insulin sensitivity (ISI) measured with hyperinsulinemic euglycemic clamp and insulin secretion using intravenous glucose tolerance test (IVGTT), as previously described (21–23). Other follow-up assessments included quarterly FPG, and annual OGTT, IVGTT and DEXA.

### 2.3 Definition of outcome measures

The primary outcome was the occurrence of prediabetes (IFG and/or IGT) or diabetes, defined by the 2003 revised American Diabetes Association criteria (15, 24, 26). For participants reaching any of those endpoints, a confirmatory test using 75-g OGTT was performed within six weeks of initial endpoint occurrence, as previously described (26). All endpoints were independently adjudicated by the Institutional Data and Safety Officer (Murray Heimberg, MD, PhD).

### 2.4 Statistical analysis

This is a *post hoc* analysis of baseline data from the POP-ABC study. Data were reported as means ± standard deviations. Differences in continuous or categorical variables between defined groups were analyzed using unpaired t test or chi square test, as appropriate. Linear regression models were used to analyze the relationship between BMD and demographic, anthropometric, glycemic, and glucoregulatory variables, and predictors of incident prediabetes were modeled using logistic regression. The annual change in BMD was analyzed using paired t test. The incidence of prediabetes across quartiles of baseline BMD was analyzed using Kaplan-Meier plots. Significance level was set as P< 0.05 (two-tailed). All analyses were performed using StatView statistical software (SAS Institute Inc., Cary, NC).

## 3 Results

### 3.1 Baseline cohort characteristics

**Figure 1:**
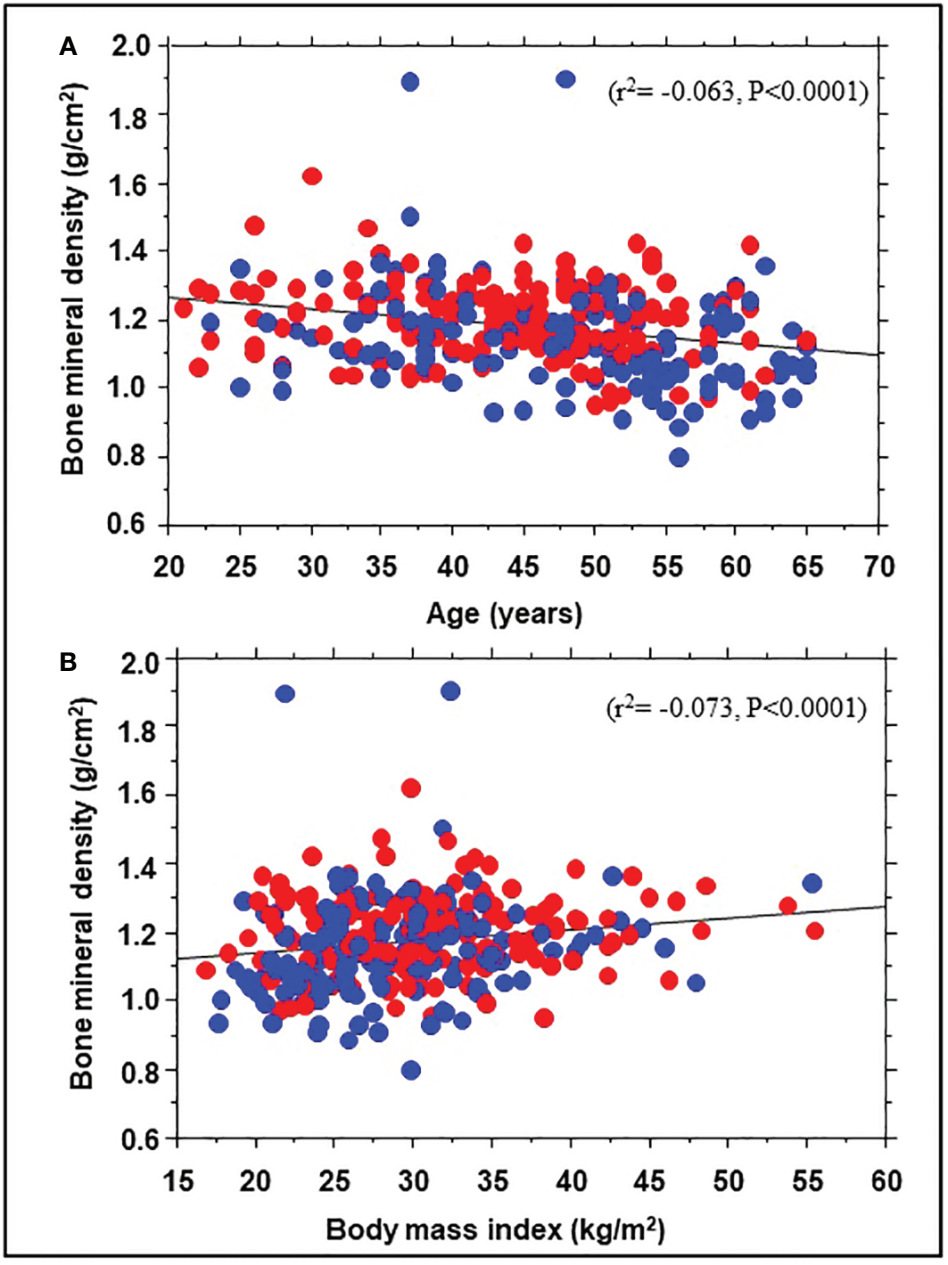
Correlation of bone mineral density with age **(A)** and body mass index **(B)** in African American (red symbols) and European American (blue symbols) participants at enrollment in the Pathobiology of Prediabetes in a Biracial Cohort study.

A total of 343 participants (193 Black, 150 White; 71% women) underwent DEXA during Year 1 of the POP-ABC study. The mean age was 44.2 ± 10.6 years; BMI was 30.2 ± 7.23 kg/m^2^. The mean FPG was 91.8 ± 6.77 mg/dl, 2hPG 124 ± 25.8 mg/dl, and HbA1c was 5.54 ± 0.44% at enrollment. The mean baseline BMD was 1.176 ± 0.135 g/cm^2^ for the entire cohort, higher in men than women (1.230 ± 0.124 g/cm^2^ vs. 1.154 ± 0.134 g/cm^2^, P<0.0001). **Table 1** shows the baseline characteristics of study subjects by ethnicity. The BMD was higher in Black vs. White participants (1.203 ± 0.114 g/cm^2^ vs. 1.146 ± 0.150 g/cm^2^, P=0.0003). Compared with White participants, African American participants had a lower mean age and higher BMI, but similar values for total and trunk fat mass (**Table 1**). Trunk fat mass and body fat mass were not significantly different by race/ethnicity. Baseline BMD was correlated inversely with age (r^2^= -0.063, P<0.0001) and directly with BMI (r^2 =^ 0.073, P<0.0001) among the Black and White POP-ABC study participants (**Figure 1**).

**Table 1:** Baseline characteristics of POP-ABC study subjects by race/ethnicity.

	African American	European American	P-Value
Number	193	150	
Age (yr)	42.5 ± 10.3	46.5 ± 10.5	0.0003
Weight (kg)	87.8 ± 21.1	81.8 ± 20.9	0.004
BMI (kg/m^2^)	31.2 ± 7.40	28.8 ± 6.78	0.0015
FPG (mg/dl)	90.8 ± 6.81	93.1 ± 6.50	0.001
2hPG (mg/dl)	123 ± 27.4	125 ± 23.3	0.45
HbA1c (%)	5.63 ± 0.47	5.44 ± 0.32	<0.0001
BMD, (g/cm^2^) Female Male	1.203 ± 0.114 1.184 ± 0.103 1.256 ± 0.127	1.146 ± 0.150 1.118 ± 0.156 1.206 ± 0.116	0.0003
Trunk fat mass(kg)	15.3 ± 7.50	14.8 ± 6.96	0.48
Total body fat mass (kg)	31.8 ± 13.76	29.4 ± 13.21	0.11
Insulin sensitivity (µmol/kg FFM.min^-1^/pM)	0.12 ± 0.07	0.14 ± 0.06	0.0352
Insulin Secretion (AIR) (µU/ml)	105 ± 88.8	61.3 ± 39.0	<0.0001

AIR, acute insulin response to i.v. glucose; BMD, bone mineral density; BMI, body mass index; AIR, acute insulin response to i.v. glucose; FPG, fasting plasma glucose; 2hPG, two-hour plasma glucose; To convert FPG and 2hPG to mmol/l, multiply by 0.56.

### 3.2 BMD and prediabetes risk

**Figure 2:**
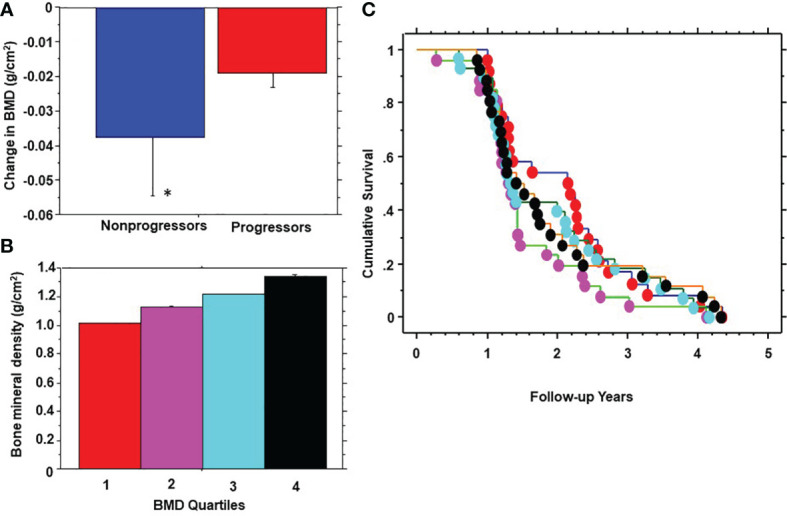
One-year change in bone mineral density (BMD) in progressors to prediabetes vs. nonprogressors **(A)**; stratification of participants by quartiles (Q) of baseline BMD **(B)**; and Kaplan-Meier plot of prediabetes survival by baseline BMD quartile **(C)** in the Pathobiology of Prediabetes in a Biracial Cohort study. BMD quartiles: 1 red, 2 purple, 3 blue, 4 black. * P<0.0001.

During 5 years of follow-up, 101 participants developed prediabetes and 10 subjects developed T2DM (progressors) and 232 maintained normoglycemia (nonprogressors). Participants who developed T2DM were not included in the present report. **Table 2** shows the demographic, clinical and biochemical characteristics of progressors to prediabetes versus nonprogressors. Compared with nonprogressors, participants who progressed to prediabetes were older, more likely to be male, and had significantly higher FPG, HbA1c, baseline BMI, and 1-year increase in BMI. Progressors also had higher insulin sensitivity and trunk fat mass but lower adiponectin levels at baseline, compared with nonprogressors (**Table 2**). Progressors to prediabetes had numerically but insignificantly higher BMD (1.177± 0.114 g/cm2 vs. 1.175 ± 0.146 g/cm^2^, P=0.88) and bone mineral content (BMC) (2.60 kg ± 0.46 vs. 2.49 kg ± 0.50, P=0.07) at baseline compared with nonprogressors. Furthermore, progressors to prediabetes experienced a significantly slower 1-year decrease in BMD compared with nonprogressors (-0.019 ± 0.46 027 vs. -0.038 ± 0.14, P<0.0001) (**Figure 2A**).

**Table 2:** Demographic, clinical and biochemical characteristics in progressors to prediabetes versus nonprogressors.

	Progressor	Nonprogressor	P value
Number	111	232	
Black/White	58/53	135/97	0.30
Women/Men	65/46	180/52	0.0003
Premenopausal/ postmenopausal	35/30	115/65	0.15
Age (yr)	47.3 ± 8.94	43.8 ± 10.8	0.0031
Weight (kg)	90.0 ± 20.1	83.1 ± 21.9	0.0051
Baseline BMI (kg/m^2^)	31.4 ± 6.88	29.6 ± 7.40	0.034
Delta BMI (1-yr) (kg/m^2^)	0.50 ± 1.48	0.16 ± 1.63	0.088
Delta BMI (2-yr) (kg/m^2^)	0.61 ± 1.85	0.30 ± 1.98	0.24
FPG (mg/dl)	94.0 ± 6.75	91.0 ± 6.49	<0.0001
HbA1c (%)	5.66 ± 0.47	5.52 ± 0.43	0.0059
BMD (g/cm^2^)	1.177± 0.114	1.175 ± 0.146	0.88
BMC (kg)	2.60 ± 0.46	2.49 ± 0.50	0.07
Delta BMD (1-yr) (g/cm^2^)	-0.02 ± 0.46	-0.04 ± 0.14	<0.0001
Trunk fat mass (kg)	16.6 ± 6.85	14.3 ± 7.34	0.0079
Total fat mass (kg)	32.0 ± 1.26	29.9 ± 1.40	0.19
Insulin sensitivity (µmol/kg FFM.min^-1^/pM)	0.12 ± 0.07	0.15 ± 0.06	0.0014
Insulin secretion (AIR) (µu/ml)	81.3 ± 73.7	88.2 ± 74.2	0.44
hsCRP (mg/L)	4.35 ± 6.67	3.55 ± 5.38	0.24
Adiponectin (µg/ml)	8.53 ± 4.35	9.87 ± 5.69	0.031

AIR, acute insulin response to i.v. glucose; BMD, bone mineral density; BMI, body mass index; FFM, fat-free mass; FPG, fasting plasma glucose; hsCRP, high sensitivity C-reactive protein; 2hPG, two-hour plasma glucose; To convert FPG and 2hPG to mmol/l, multiply by 0.56.

In logistic regression models, BMC and BMD z score significantly predicted incident prediabetes, after adjusting for age, BMI, change in BMI, ethnicity, FPG, 2hPG, total fat mass and trunk fat mass, and adiponectin at enrollment. More negative BMD z scores (indicating lower bone mass referenced to age- and sex-matched control) were associated with decreased risk of incident prediabetes (adjusted odds ratio 0.598 [95% confidence interval 0.407 - 0.877], P=0.0085). In contrast, higher BMC at baseline predicted increased risk of incident prediabetes (adjusted odds ratio 1.001[95% confidence interval 1.000 – 1.002], P=0.0052).

We stratified participants by quartiles of baseline BMD (**Figure 2B**) and analyzed the development of prediabetes across BMD strata (**Figure 2C**). From Kaplan-Meier analysis, the time to 50% prediabetes survival was 2.15 years among participants with the lowest BMD at baseline (Quartile 1) versus 1.31 – 1.41 years among subjects in higher BMD quartiles.

### 3.3 Potential underlying mechanisms

**Figure 3:**
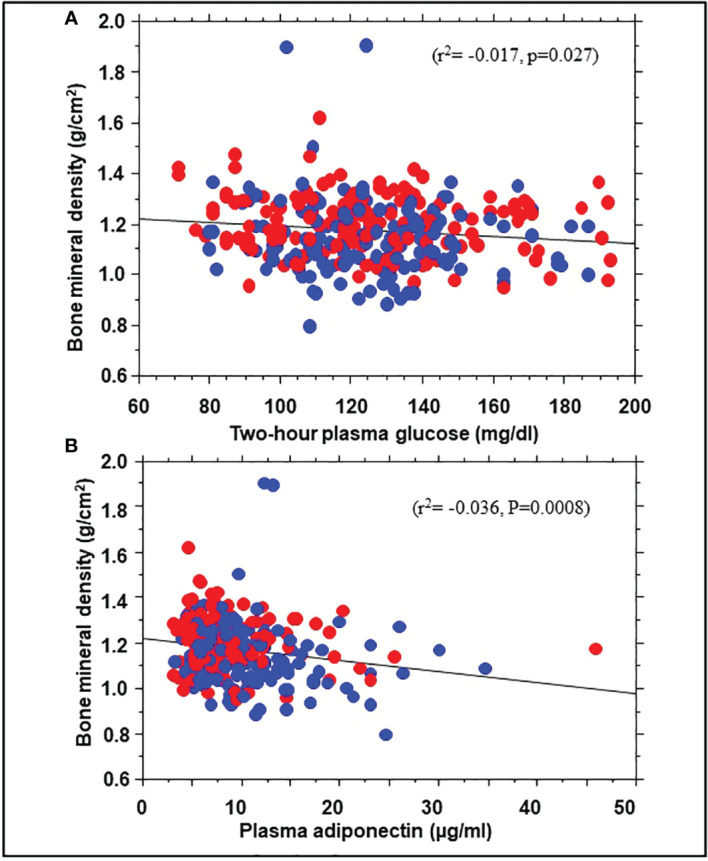
Correlation of bone mineral density with 2hPG **(A)** and adiponectin levels **(B)** in African American (red symbols) and European American (blue symbols) participants at enrollment in the Pathobiology of Prediabetes in a Biracial Cohort study.

To explore possible mechanisms for the association of BMD with incident prediabetes, we examined the relationship between BMD and several baseline variables. Univariate linear regression showed significant correlations between BMD and body weight (r^2 =^ 0.10, P<0.0001), BMI (r^2 =^ 0.029, P=0.0028), total body fat mass (r^2 =^ 0.044, p=0.0003), trunk fat mass (r^2 =^ 0.033, P= 0.0021), 2hPG (r^2^= -0.017, p=0.027), and adiponectin levels (r^2^= -0.036, P=0.0008) but not FPG, HbA1c, insulin sensitivity, insulin secretion, or C-reactive protein (**Figure 3**). A multivariate regression model was run, with BMD as dependent variable and BMI along with 2hPG, adiponectin levels, C-reactive protein, insulin sensitivity and insulin secretion as independent variables. The significant predictors of BMD were BMI (beta coefficient 0.18, P=0.05), 2hPG (beta coefficient -0.16, P=0.028), and adiponectin (beta coefficient -0.20, P=0.0098).

## 4 Discussion

In our prospective study of healthy offspring of parents with T2DM, bone density at enrollment had the expected relationships with age, sex, and ethnicity. Study participants who developed incident prediabetes during 5 years of follow-up tended to have higher baseline BMD and BMC and showed a significantly slower 1-year decline in BMD compared with nonprogressors. After controlling for baseline variables (including age, BMI, and blood glucose), higher bone mass predicted increased 5-year risk of progression from normoglycemia to prediabetes. These findings suggest an inverse relationship between baseline bone mass and incident prediabetes risk.

Previous cross-sectional studies had reported higher BMD in people with T2DM compared with individuals without diabetes (4–6). The findings from our prospective POP-ABC study demonstrate a similar association between BMD and prediabetes, consistent with previous findings from cross-sectional surveys (19, 20). Exploring possible mechanisms, we observed significant correlations between baseline BMD and measures of adiposity and glucose tolerance (2hPG) among our study participants. However, our findings associating higher bone mass with incident prediabetes risk persisted after adjusting for adiposity and glycemia. No significant associations were observed between BMD and insulin sensitivity, insulin secretion, or hsCRP levels in our POP-ABC participants, all of whom were normoglycemic at baseline.

The link between higher BMD and increased prediabetes risk requires further mechanistic insights. The higher BMD reported in people with T2DM could be explained at least in part by obesity. The association of BMD with adiposity measures in our present study also supports a role for obesity as a contributing factor for the higher BMD in progressors versus nonprogressors to prediabetes. Besides obesity, hyperglycemia, insulin resistance, or hyperinsulinemia might be possible mediators of increased bone density in people with T2DM and prediabetes (5–7, 27). As a corollary, the lower BMD reported in people with T1DM would be consistent with the underlying beta-cell failure and insulin deficiency (10). Insulin stimulates osteoblast formation and promotes proliferation, differentiation, and survival of osteoblasts, with an overall balance in favor of bone formation (10). Thus, the relative hyperinsulinemia observed in insulin-resistant individuals with obesity, T2DM, and prediabetes would favor accrual of bone mass, although the effect may be modified by the severity of insulin resistance and ambient adipocytokines (4–7, 27, 28).

Plasma adiponectin levels were lower in progressors to prediabetes compared with nonprogressors, and inversely correlated with BMD in our study cohort. Adiponectin, the most abundant secreted product of adipocytes, is a beneficial marker of cardiometabolic health that has been associated with decreased risks of development of diabetes and progression from prediabetes T2DM (29, 30). In a previous report from the POP-ABC study, lower baseline adiponectin levels predicted higher risk of progression from normoglycemia to prediabetes (31). Taken together, our findings of lower baseline adiponectin levels in progressors to prediabetes versus nonprogressors, an inverse correlation between adiponectin and BMD, and a positive association between BMD and incident prediabetes, implicate adiponectin as a possible mediator of the link between BMD and prediabetes risk. Previous reports have also shown a negative correlation between adiponectin and BMD (32, 33). The mechanisms underlying the negative association between adiponectin and BMD are unclear, but increased bone marrow adipogenesis with associated increase in adiponectin production has been proposed to mediate decreased BMD (34, 35).

In addition to the mechanisms involving insulinemia and adiponectin on BMD, there might be a possible mechanism linking bone metabolism to dysglycemia *via* osteocyte production of sclerostin, an inhibitor of wnt signaling pathway. The possible metabolic effects of inhibiting wnt signaling pathway include downstream consequences on adipogenesis, TCF7L2 gene expression, incretin processing and glucose dysregulation (36–39). Another putative mechanism might involve osteocalcin. In a recent study of 240 women with prior gestational diabetes mellitus, participants with prediabetes or diabetes tended to have higher BMD and significantly lower serum osteocalcin levels compared with normoglycemic control (40). Osteocalcin levels declined serially as glycemic status shifted from normoglycemia to prediabetes to diabetes, and showed significant associations with BMD, plasma glucose, insulin sensitivity and insulin secretion in the study population (40).

The strengths of study include the prospective design, enrolment of a diverse study cohort, and the use of robust methodologies for assessment of prediabetes endpoints, insulin sensitivity and insulin secretion. Despite these strengths, our study has some limitations. First, the associations between BMD and prediabetes risk, and the related mechanisms that we observed, do not indicate causality. Second, we studied a special population (offspring of T2DM parents), which may limit the extrapolation of our findings to the general population of individuals without a family history of T2DM. Third, we used fasting plasma glucose and 2-hour OGTT plasma glucose values for definition of prediabetes and did not include HbA1c as one of the criteria. Thus, we may have underdiagnosed individuals with normal fasting and 2-hour plasma glucose values but prediabetes-range HbA1c levels. Fourth, we did not assess vitamin D level, bone micro-architecture, or bone turnover markers.in our participants. Vitamin D deficiency has been associated with increased risks of T2DM and prediabetes (41, 42). However, vitamin D deficiency leads to osteomalacia and decreased bone density (43). Thus, our present finding of an association between higher bone density and increased risk of prediabetes is not likely explained by mechanisms involving vitamin D status (44). Furthermore, our conclusions based on baseline assessments do not account for possible temporal changes in those parameters that might have occurred during the follow-up period. In conclusion, our prospective study demonstrates that the previously reported association between higher bone density and T2DM is discernible in people with prediabetes risk. Thus, putative mechanisms linking bone metabolism with dysglycemia could be operational long before the occurrence of clinical diabetes. Thus, our findings suggest that BMD might be a biomarker for incident glycemic deterioration among normoglycemic individuals.

## Data availability statement

The raw data supporting the conclusions of this article will be made available by the authors, without undue reservation.

## Ethics statement

The studies involving human participants were reviewed and approved by University of Tennessee Institutional Review Board. The patients/participants provided their written informed consent to participate in this study.

## Author contributions

SD-J, as the principal investigator, developed the study concept and design, analyzed data, and wrote the manuscript; ZL collected data, reviewed and revised the manuscript; PA collected data, reviewed and revised the manuscript; AP collected data, reviewed and revised the manuscript, JW analyzed data, reviewed and revised the manuscript. All authors contributed to the article and approved the submitted version.
